# Sedentary behaviour in hospitalised older people: a scoping review protocol

**DOI:** 10.1186/s13643-020-01290-0

**Published:** 2020-02-19

**Authors:** Unyime S. Jasper, Lalit Yadav, Agathe Daria Jadczak, Solomon Yu, Renuka Visvanathan, Joanne Dollard

**Affiliations:** 1 Adelaide Geriatrics Training and Research with Aged Care (G-TRAC) Centre, Adelaide Medical School, Faculty of Health and Medical Sciences, University of Adelaide, Adelaide, SA Australia; 2 Basil Hetzel Institute for Translational Research, 37 Woodville Road, Woodville, Adelaide, 5011 Australia; 3 National Health and Medical Research Council Centre of Research Excellence: Frailty Trans-Disciplinary Research to Achieve Healthy Ageing, University of Adelaide, Adelaide, SA Australia; 4 Aged and Extended Care Services, The Queen Elizabeth Hospital, Central Adelaide Local Health Network, Adelaide, SA Australia

**Keywords:** Sedentary behaviour, Older people, Hospital or acute care, Scoping review, Perception, Intervention

## Abstract

**Background:**

Older adults spend up to 23 h daily sitting or lying while in hospital. Sedentary behaviour (SB) within a hospital setting is often associated with poor health outcomes including physical and cognitive decline, reduced quality of life and death as well as hospital readmissions. Conversely, replacing SB with mild to moderate levels of physical activity such as walking can significantly reduce hospital readmission risk by 30 days. Given the potentially harmful effects of SB in hospitalised older adults, it is vital to identify current literature by broadly exploring different aspects of SB among older people in hospital. The overall aim of this scoping review is to produce a literature map of current evidence on key domains of sedentary behaviour in hospitalised older people.

**Method:**

A search for relevant publications will be undertaken in Pedro, MEDLINE Ovid, Cochrane, Scopus, Cumulative Index to Nursing and Allied Health Literature, PsychInfo, Embase, Ageline, Joanna Briggs Institute (JBI) and clinical trials registries. Publications in English and those where the author can provide the full text in English will be included. Studies conducted in hospitals (including in-patient rehabilitation facilities) or acute and subacute care settings and in people aged ≥ 65 will be included. A three-stage method will be used to identify relevant articles, consisting of database search using keywords, keywords and index words across all databases, and reference searching. Articles will be selected following screening of titles/abstracts succeeded by a full-text appraisal utilising a standardised selection form. Two independent reviewers will extract data using the standardised form that will be tested on two articles. A narrative summary will accompany results presented in tables and figures.

## Background

Sedentary behaviour (SB), according to the Sedentary Behaviour Research Network, is any low energy expenditure [≤ 1.5 Metabolic Equivalent of Task (MET)] behaviour undertaken when sitting or lying whilst awake [1]. Owing to its critical association with health, SB has gained increasing attention over the last decade [2]. Sedentary behaviour bouts (SBBs) are described as “one or more consecutive minutes with less than 100 step counts/minute” whilst sedentary time is the total time spent being sedentary during waking hours [3]. SBB provides insight into the duration and time of day hospitalised older people are sedentary, and this information can be utilised when planning interventions to break sedentary time in hospitalised older people.

SB in older people has been linked with diseases such as Alzheimer’s disease and other dementia, cardiovascular disease, type 2 diabetes, cancer and osteoporosis, poor health outcomes such as physical and cognitive decline, reduced quality of life and wellbeing, depression and frailty, increased risk of premature death and mortality and hospital use such as readmissions [4–6]. For older hospitalised people, there is an association between SB and poor recovery following admission resulting in mortality, longer length of hospital stay and readmissions [7, 8]. Despite these negative consequences, SB is very common with older hospitalised people spending up to 23 h daily sitting or lying [9].

Given the harmful effects of SB, it is vital to explore current evidence on SB and SBB among older people to inform practice, research and future interventions. This will be restricted to the hospital setting because of the high prevalence of SB and the potentially significant negative impact of this on hospitalised older people [9, 10]. Furthermore, while there are some assessment and intervention research about SB involving community-dwelling older people [4], there is a dearth of similar research in hospital settings, where the issue has long been recognised as a problem [11]. The negative associations between SB and health outcomes and increased hospital use highlight the urgent need to decrease SB among hospitalised older people.

A preliminary search revealed that there were no existing scoping review protocols or finalised systematic/scoping reviews on SB in hospitalised older people. A search was conducted in Medline on 7 July 2019. To date, there has been one systematic review on the epidemiology of SB and SBB among hospitalised adult patients [7]. However, it only included studies that utilised objective assessments (accelerometry) of SB, excluded subjective assessments of SB, did not focus on older adults and excluded studies in subacute settings. Another systematic review focused on epidemiology, assessment and interventions of SB in older hip fracture patients but excluded older patients with medical and other orthopaedic conditions, subjective assessments and SBBs [11]. Therefore, seeking evidence on SB and SBB in the hospital setting warrants further exploration. Thus, the primary aim of this review is to describe the state of the peer-reviewed literature on key domains of SB in hospitalised older people

## Methods

### Study design

To map the range, scope and types of studies available on the topic of interest, a scoping review will be undertaken. The methodology, as well as stages for this scoping review, will be guided by the Joanna Briggs Institute (JBI)’s “methodology for scoping reviews” [12]. The final output will adhere to the Preferred Reporting Items for Systematic Reviews and Meta-Analysis extension for Scoping Reviews (PRISMA-ScR) checklist [13]. Accordingly, this review protocol was developed using the PRISMA-Protocols (PRISMA-P) 2015 checklist [14] (Additional file 1)

According to Colquhoun et al ([15]: 18), a scoping review is “a form of knowledge synthesis that addresses an exploratory research question aimed at mapping key concepts, types of evidence and gaps in research related to a defined area or field by systematically searching, selecting and synthesising existing knowledge.” Thus, we will undertake a scoping review because it enables the exploration of general questions and provides an overview of a topic rather than an in-depth synthesis of a limited question [16]. This is especially the case for an emerging topic such as SB and SBB in hospitalised older adults, where information has not been comprehensively reviewed and is diverse [17]. A scoping review allows the mapping of research carried out so far in the field of SB and SBB in hospitalised older people, to identify and analyse knowledge gaps that are important for future research [12] and may serve as a prerequisite to a systematic review and clinical studies [12]. Thus, this scoping review will identify and produce a literature map of key domains of SB in hospitalised older people.

### Eligibility criteria

#### Population/studies

Inpatients aged 65 years and older (or where the mean age is > 65 years), their carers and health professionals will be included. Health professionals refer to medical, nursing and allied health professionals involved in the care of older people.

#### Concept

Inclusion criteria include:

- Prevalence rates of SB and SBB in hospitalised older people measured objectively or subjectively;
- Tools used to assess SB and SBB in hospitalised older people, including objective and subjective measures;
- Intervention strategies and outcomes for reducing SB and SBB in hospital; and
- Patient, carer and health professional experience, perspective or outcomes of intervention strategies to reduce SB and SBBs while in hospital (including mixed methods, qualitative and quantitative studies).

#### Context

All studies on SB and SBB conducted in hospitals without being limited to geographic region, ethnicity or gender, will be included. Hospital in this review includes acute and subacute (such as rehabilitation and geriatric evaluation and management units) settings.

#### Study types

This scoping review will review published primary research studies using mixed methods, quantitative and qualitative methodology. Studies identified during the literature search that focused on physical activity but reported data on SB or SBBs will be included in this review where they meet the rest of the inclusion criteria.

Exclusion criteria include:

- Studies involving adults aged < 65 years (or where the mean age < 65 years);
- Studies focused on older people living in the community or nursing homes or aged care.

#### Search strategy

The search strategy aims to identify studies published in English since 2001 (when the first guidelines for reducing SB was published in Canada) [18]. The search will be conducted from July 07, 2019, until the review is accepted for publication. Studies published in other languages (apart from English) will be included if the authors can provide an English version on request.

The search strategy will be developed in MEDLINE utilising medical subject headings (MeSH) and text keywords. A health sciences academic librarian will review the keywords. To identify relevant literature, a three-step search methodology will be employed. Firstly, databases will be searched using keywords (example attached in Additional file 2). Then, the text words contained in the title and abstract will be analysed as well as the index words describing the article. The second search will involve the use of all the keywords and index words cross all databases. In the third step, the bibliography of every identified article and reports will be thoroughly searched for additional studies. The databases that will be searched include Pedro, MEDLINE Ovid, Cochrane, Scopus, Cumulative Index to Nursing and Allied Health Literature (CINAHL), PsychInfo, Embase, Ageline, JBI and clinical trials registries for interventions. All duplicates will be removed when the publications are screened.

### Data extraction

The methodology of scoping reviews developed by the JBI will be used to extract data from all the articles that will be included in this scoping review [12]. A data extraction form that is in line with the research aim and objectives of this study will be used to chart the extracted data (see Additional file 3). Nevertheless, process of data extraction may be further refined as the review progresses.

Two reviewers will work independently to extract data, and the extracted data will be charted into a Microsoft excel form as shown in Additional file 3. If there are disagreements between the reviewers, they will discuss the differences, and a third reviewer will be invited if they cannot reach a consensus. The reviewers will test the data extraction form using two studies. The knowledge gained from this will then be used to modify and revise the data extraction form as required. These modifications will be outlined in the complete scoping review paper. Two independent reviewers will assess the full text of selected citations detail against the inclusion criteria. Reasons for exclusion of full text studies that did not meet the inclusion criteria will be recorded and reported in the scoping review. The final paper will contain a PRISMA flow diagram showing the search and screening processes.

### Data management

References retrieved from the various electronic database will be managed with EndNote 8® software. With EndNote 8®, it will be able to easily find and extract duplicates; conduct, effectively superintend and have a clear trail of the document screening process; classify articles which will be included or excluded from the bibliometric review, and efficiently manage the full text versions of articles to be included. The completed data extraction form will also be attached to the Endnote 8® library.

### Presentation of the results/data mapping

The data will be presented in a table or as a diagram in line with the aim of this review. The results will be presented in a tabular format, followed by a narrative summary describing how the results relate to the review aim (see Additional file 4). However, during the review process, this table may be refined as necessary.

## Discussion

Since the concept of understanding SB among older people in hospital is relatively new, a scoping review is the best approach, because it will ensure that the literature covered will be as broad as possible. This scoping review will identify and map evidence on SB and SBB among older people in hospital. It is expected that the findings of this study will provide clear and in-depth evidence of the extent of SB among older people in hospital, acute and sub-acute care as well current methods used to assess SB and SBB. Furthermore, strategies that have been employed thus far to reduce SB and SBB among older people in hospital will be described. The acceptability of such interventions as perceived by stakeholders will be clarified. Knowing these will help inform the design of future research targeting gaps in the evidence. There is evidence that reducing SB and SBB could contribute to increased levels of physical activity among older people in hospital, which in turn has been linked to better functional recovery, quality of life, improved mobility and activities of daily living at discharge and 4 months after [19]. We anticipate that the review will be useful to a variety of stakeholders who have an interest in reducing SB and SBBs to promote the desired recovery among older people in hospital.

## Supplementary information

**Additional file 1.** PRISMA-P 2015 Checklist.

**Additional file 2.** CINAHL search.

**Additional file 3.** Data extraction form.

**Additional file 4.** Data presentation table for SB and SBBR2.

## Data Availability

Data sharing is not applicable to this article because no datasets were generated or analysed for the study.

## Abbreviations

SB: Sedentary behaviour
