# Supplementary material for: Sedentary behaviour in hospitalised older people: a scoping review protocol
Source: Syst Rev. 2020 Feb 19;9:36. doi: 10.1186/s13643-020-01290-0 (PMC7031934; doi:10.1186/s13643-020-01290-0)
Supplement: Supplementary file 2 — Additional file 2. CINAHL search. [file 13643_2020_1290_MOESM2_ESM.pdf]

Wednesday, January 15, 2020 3:35:41 AM

#	Query	Limiters/Expanders	Last Run Via	Results
S17	((hospital* or acute setting* or inpatient* or ward*) AND (S12 OR S13 OR S14)) AND (S7 AND S11 AND S15)	Limiters - Published Date: 20010101-20201231 Expanders - Apply equivalent subjects Search modes - Find all my search terms	Interface - EBSCOhost Research Databases Search Screen - Advanced Search Database - CINAHL with Full Text	268
S16	((hospital* or acute setting* or inpatient* or ward*) AND (S12 OR S13 OR S14)) AND (S7 AND S11 AND S15)	Expanders - Apply equivalent subjects Search modes - Find all my search terms	Interface - EBSCOhost Research Databases Search Screen - Advanced Search Database - CINAHL with Full Text	312
S15	(hospital* or acute setting* or inpatient* or ward*) AND (S12 OR S13 OR S14)	Expanders - Apply equivalent subjects Search modes - Find all my search terms	Interface - EBSCOhost Research Databases Search Screen - Advanced Search Database - CINAHL with Full Text	556,493
S14	hospital* or acute setting* or inpatient* or ward*	Expanders - Apply equivalent subjects Search modes - Find all my search terms	Interface - EBSCOhost Research Databases Search Screen - Advanced Search Database - CINAHL with Full Text	556,493
S13	(MH "Inpatients")	Expanders - Apply	Interface -	76,511

		equivalent subjects Search modes - Find all my search terms	EBSCOhost Research Databases Search Screen - Advanced Search Database - CINAHL with Full Text	
S12	(MH "Hospitals")	Expanders - Apply equivalent subjects Search modes - Find all my search terms	Interface - EBSCOhost Research Databases Search Screen - Advanced Search Database - CINAHL with Full Text	53,489
S11	(geriatric or older adult or elderly or senior or elderly people or older or older people) AND (S8 OR S9 OR S10)	Expanders - Apply equivalent subjects Search modes - Find all my search terms	Interface - EBSCOhost Research Databases Search Screen - Advanced Search Database - CINAHL with Full Text	275,964
S10	geriatric or older adult or elderly or senior or elderly people or older or older people	Expanders - Apply equivalent subjects Search modes - Find all my search terms	Interface - EBSCOhost Research Databases Search Screen - Advanced Search Database - CINAHL with Full Text	275,964
S9	old* (age* OR people OR person* OR patient*)	Expanders - Apply equivalent subjects Search modes - Find all my search terms	Interface - EBSCOhost Research Databases Search Screen - Advanced Search Database - CINAHL with Full Text	270,410
S8	(MH "Aged")	Expanders - Apply	Interface -	731,553

		equivalent subjects Search modes - Find all my search terms	EBSCOhost Research Databases Search Screen - Advanced Search Database - CINAHL with Full Text	
S7	(sitting OR seated OR lying OR reclining position OR sedentary behavio#* OR (sedentary lifestyle* or sedentary behavior or inactivity) OR MH("Sitting") OR (MH "Life Style, sedentary")) AND (S1 OR S2 OR S3 OR S4 OR S5)	Expanders - Apply equivalent subjects Search modes - Find all my search terms	Interface - EBSCOhost Research Databases Search Screen - Advanced Search Database - CINAHL with Full Text	27,311
S6	(sitting OR seated OR lying OR reclining position) OR sedentary behavio#* OR (sedentary lifestyle* or sedentary behavior or inactivity) OR MH("Sitting") OR (MH "Life Style, sedentary")	Expanders - Apply equivalent subjects Search modes - Find all my search terms	Interface - EBSCOhost Research Databases Search Screen - Advanced Search Database - CINAHL with Full Text	27,311
S5	sitting OR seated	Expanders - Apply	Interface -	13,991

	OR lying OR reclining position	equivalent subjects Search modes - Find all my search terms	EBSCOhost Research Databases Search Screen - Advanced Search Database - CINAHL with Full Text	
S4	sedentary behavio#*	Expanders - Apply equivalent subjects Search modes - Find all my search terms	Interface - EBSCOhost Research Databases Search Screen - Advanced Search Database - CINAHL with Full Text	5,571
S3	sedentary lifestyle* or sedentary behavior or inactivity	Expanders - Apply equivalent subjects Search modes - Find all my search terms	Interface - EBSCOhost Research Databases Search Screen - Advanced Search Database - CINAHL with Full Text	12,181
S2	MH("Sitting")	Expanders - Apply equivalent subjects Search modes - Find all my search terms	Interface - EBSCOhost Research Databases Search Screen - Advanced Search Database - CINAHL with Full Text	2,447
S1	(MH "Life Style, sedentary")	Expanders - Apply equivalent subjects Search modes - Find all my search terms	Interface - EBSCOhost Research Databases Search Screen - Advanced Search Database - CINAHL with Full Text	7,156
