# Supplementary material for: Sedentary behaviour in hospitalised older people: a scoping review protocol
Source: Syst Rev. 2020 Feb 19;9:36. doi: 10.1186/s13643-020-01290-0 (PMC7031934; doi:10.1186/s13643-020-01290-0)
Supplement: Supplementary file 3 — Additional file 3. Data extraction form. [file 13643_2020_1290_MOESM3_ESM.docx]

**Table 2: Data extraction form**

Reviewer:

Date of data extraction:

**Data to be extracted Item Notes to reviewer**

Publication details

- Authors

Year

Article title

Journal, volume, issue, page numbers

Study design and details

- Setting (country; characteristics of hospital/acute care/rehabilitation)
- Sampling technique (convenience sampling; probability sampling)
- Sample size
- Details of study participants (age, sex, disease condition, etc.)
- Design (Survey; Randomised Control Trials (RCTs), etc.)
- Method of data collection (interviews; observation, etc.)
- Method of data analysis

Specific details of interest for the scoping review

- SB tools used (subjective; objective, etc.): name of tool,

abbreviation of the tool, details of the tool;

make of the tool (if objective)

- SBB tools used (subjective; objective, etc.): name of tool,

abbreviation of the tool, details of the tool;

make of the tool (if objective)

- Reported prevalence rates of SB and SBB
- Reported treatment modalities for SB and SBB
- Reported outcomes of the treatment of SB and SBB
- Geriatric specific outcomes reported as benefitting

from treatment of SB and SBB

- Reported patients, carers and health professionals perception

of intervention to reduce SB and SBB
