# Supplementary material for: Sedentary behaviour in hospitalised older people: a scoping review protocol
Source: Syst Rev. 2020 Feb 19;9:36. doi: 10.1186/s13643-020-01290-0 (PMC7031934; doi:10.1186/s13643-020-01290-0)
Supplement: Supplementary file 4 — Additional file 4. Data presentation table for SB and SBBR2. [file 13643_2020_1290_MOESM4_ESM.docx]

**Data presentation (mapping table): epidemiology of SB and SBB**

| Study design  Country | Number of subjects  Setting  Recruitment strategy | Prevalence of SB  Prevalence of SBB | Health outcomes associated with SB and SBB  Mobility  Gait speed  Function  Quality of life  Wellbeing  Cognition | Limitations of study  Identified gaps in evidence |
| --- | --- | --- | --- | --- |

**Data presentation (mapping table): assessment of SB and SBB**

| Study design  Country | Number of subjects  Setting  Recruitment strategy | Assessment methods for SB  Assessment methods for SBB | Limitations of study  Identified gaps in evidence |
| --- | --- | --- | --- |

**Data presentation (mapping table): interventions on SB and SBB**

| Study design  Country | Number of subjects  Setting  Recruitment strategy | Interventions on SB  Interventions on SBB | Health outcomes associated with SB and SBB  Mobility  Gait speed  Function  Quality of life  Wellbeing  Cognition | Limitations of study  Identified gaps in evidence |
| --- | --- | --- | --- | --- |

**Data presentation (mapping table): staff perception of intervention**

| Study design  Country | Number of subjects  Setting  Recruitment strategy | Description of intervention  Perception of intervention  Suggestions for future intervention | Limitations of study  Identified gaps in evidence |
| --- | --- | --- | --- |

**Data presentation (mapping table): patient and carers perception of intervention**

| Study design  Country | Number of subjects  Setting  Recruitment strategy | Description of intervention  Perception of intervention  Suggestions for future intervention | Limitations of study  Identified gaps in evidence |
| --- | --- | --- | --- |
